# Cell Populations Expressing Stemness-Associated Markers in Vascular Anomalies

**DOI:** 10.3389/fsurg.2020.610758

**Published:** 2021-02-09

**Authors:** Ethan J. Kilmister, Lauren Hansen, Paul F. Davis, Sean R. R. Hall, Swee T. Tan

**Affiliations:** ^1^Gillies McIndoe Research Institute, Wellington, New Zealand; ^2^Wellington Regional Plastic, Maxillofacial and Burns Unit, Hutt Hospital, Wellington, New Zealand; ^3^Department of Surgery, The Royal Melbourne Hospital, The University of Melbourne, Melbourne, VIC, Australia

**Keywords:** vascular anomalies, vascular tumor, vascular malformation, embryonic stem cells, induced pluripotent stem cells, renin-angiotensin system, gene mutations, stemness-associated markers

## Abstract

Treatment of vascular anomalies (VAs) is mostly empirical and, in many instances unsatisfactory, as the pathogeneses of these heterogeneous conditions remain largely unknown. There is emerging evidence of the presence of cell populations expressing stemness-associated markers within many types of vascular tumors and vascular malformations. The presence of these populations in VAs is supported, in part, by the observed clinical effect of the mTOR inhibitor, sirolimus, that regulates differentiation of embryonic stem cells (ESCs). The discovery of the central role of the renin-angiotensin system (RAS) in regulating stem cells in infantile hemangioma (IH) provides a plausible explanation for its spontaneous and accelerated involution induced by β-blockers and ACE inhibitors. Recent work on targeting IH stem cells by inhibiting the transcription factor SOX18 using the stereoisomer R(+) propranolol, independent of β-adrenergic blockade, opens up exciting opportunities for novel treatment of IH without the β-adrenergic blockade-related side effects. Gene mutations have been identified in several VAs, involving mainly the PI3K/AKT/mTOR and/or the Ras/RAF/MEK/ERK pathways. Existing cancer therapies that target these pathways engenders the exciting possibility of repurposing these agents for challenging VAs, with early results demonstrating clinical efficacy. However, there are several shortcomings with this approach, including the treatment cost, side effects, emergence of treatment resistance and unknown long-term effects in young patients. The presence of populations expressing stemness-associated markers, including transcription factors involved in the generation of induced pluripotent stem cells (iPSCs), in different types of VAs, suggests the possible role of stem cell pathways in their pathogenesis. Components of the RAS are expressed by cell populations expressing stemness-associated markers in different types of VAs. The gene mutations affecting the PI3K/AKT/mTOR and/or the Ras/RAF/MEK/ERK pathways interact with different components of the RAS, which may influence cell populations expressing stemness-associated markers within VAs. The potential of targeting these populations by manipulating the RAS using repurposed, low-cost and commonly available oral medications, warrants further investigation. This review presents the accumulating evidence demonstrating the presence of stemness-associated markers in VAs, their expression of the RAS, and their interaction with gene mutations affecting the PI3K/AKT/mTOR and/or the Ras/RAF/MEK/ERK pathways, in the pathogenesis of VAs.

## Introduction

Vascular anomalies (VAs) consist of a heterogenous group of disorders with infantile hemangioma (IH) being the most common, affecting 4–10% of infants (1). VAs may cause disfigurement and/or functional problems (2).

There has been a recent paradigm shift in the treatment of IH using β-blockers (3, 4) and angiotensin-converting enzyme (ACE) inhibitors (5, 6). One proposed mechanism of these agents is by targeting stem cells by modulating the renin-angiotensin system (RAS) (1, 7). For most other VAs, treatment remains empirical and unsatisfactory, although sirolimus (rapamycin), an mTOR inhibitor, is increasingly used for complex vascular anomalies (8).

Nomenclature applied to VAs has confused clinicians and patients, leading to incorrect diagnosis, improper treatment and misdirected research efforts (9). In 1982, Mulliken and Glowacki (10) first proposed a biologic classification for VAs that differentiates (infantile) hemangioma from vascular malformations. IH is characterized by an initial postnatal rapid proliferation followed by a slow involution, in which the cellular elements are gradually replaced by fibrofatty tissues (10). Vascular malformations are present at birth, grow commensurately throughout childhood and do not regress (10).

**Figure 1:**
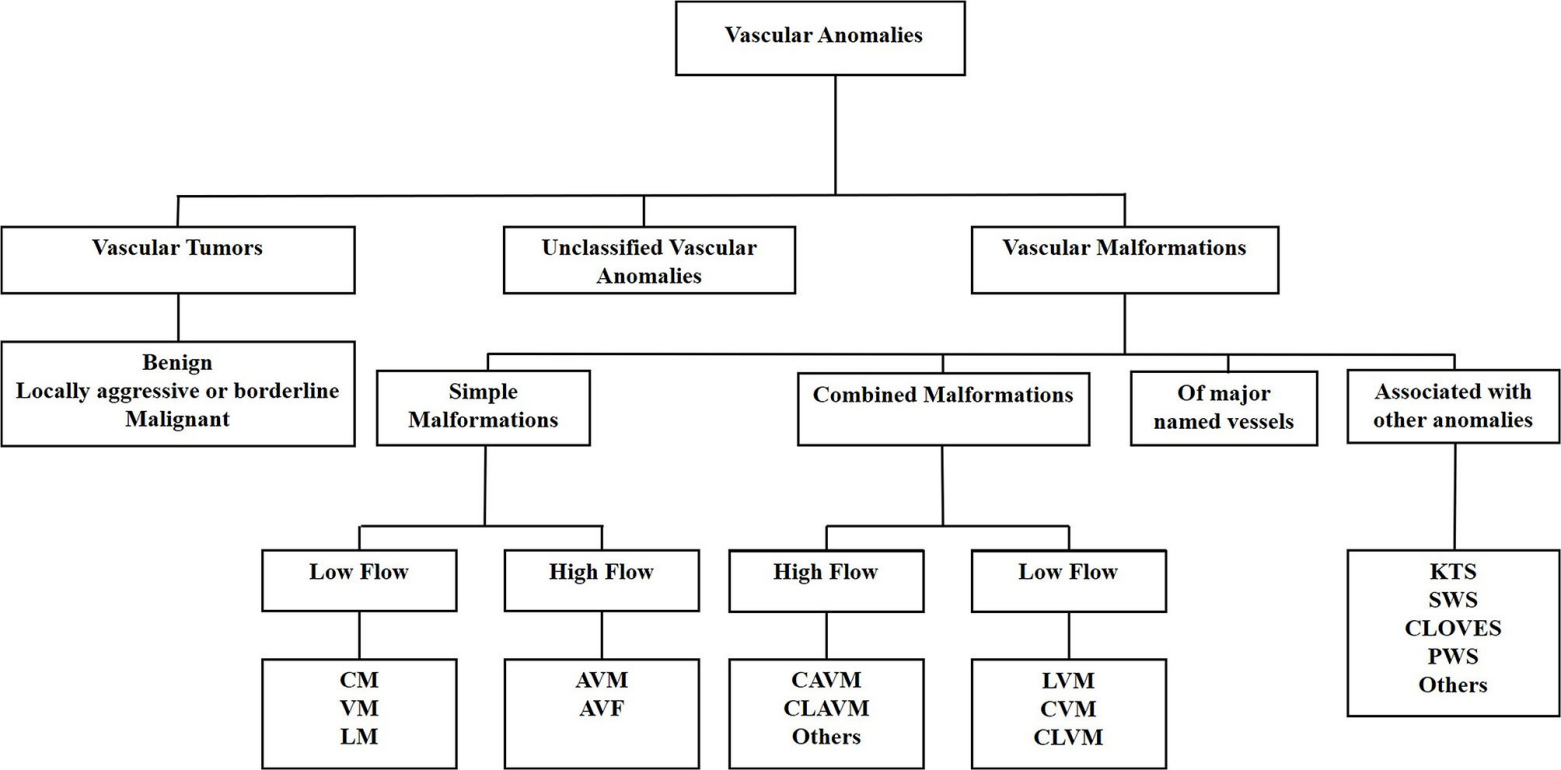
The International Society for the Study of Vascular Anomalies classification of vascular anomalies. Vascular anomalies (VAs) are categorized as vascular tumors, vascular malformations and unclassified VAs. Vascular tumors are categorized as being benign, locally aggressive or borderline, or malignant. Vascular malformations are categorized as simple malformations, combined malformations, of major named vessels, or are associated with other anomalies. Simple and combined malformations can be either high- or low-flow. AVF, arterio-venous fistula; AVM, arterio-venous malformation; CAVM, capillary-arteriovenous malformation; CLAVM, capillary-lymphatic-arteriovenous malformation; CLOVES, congenital lipomatous overgrowth-vascular malformation—epidermal nevi-spinal anomaly syndrome; CLVM, capillary-lymphatic-venous malformation; CM, capillary malformation; CVM, capillary-venous malformation; KTS, Klippel-Trénaunay syndrome; LM, lymphatic malformation; LVM, lymphatic venous malformation; PWS, port-wine stain; SWS, Sturge-Weber syndrome; VM, venous malformation.

A biologic classification of VAs was created by the International Society for the Study of Vascular Anomalies in 1997, categorizing VAs into vascular tumors and vascular malformations (11). Increasing knowledge of VAs led to a revision of the classification in 2014 which further subcategorizes vascular tumors into benign, locally aggressive or borderline, and malignant tumors. Vascular malformations are categorized as simple malformations which may be high-flow or low-flow (2), combined, of major named vessels, or those associated with other anomalies (9) (Figure 1). However, the term *hemangioma* continues to be erroneously applied to different types of VAs, despite them being distinct biological entities (12).

This review discusses accumulating evidence implicating a role for populations of cells that express stemness-associated markers, the RAS, and the influence of gene mutations on the pathobiology of VAs, underscoring commonalities between VAs. This includes a potential stem cell origin, genetic mutations, treatment options such as sirolimus, and newer targeted therapies in development and their potential shortcomings.

### Gene Mutations in Vascular Anomalies and Overgrowth Syndromes

**Figure 2:**
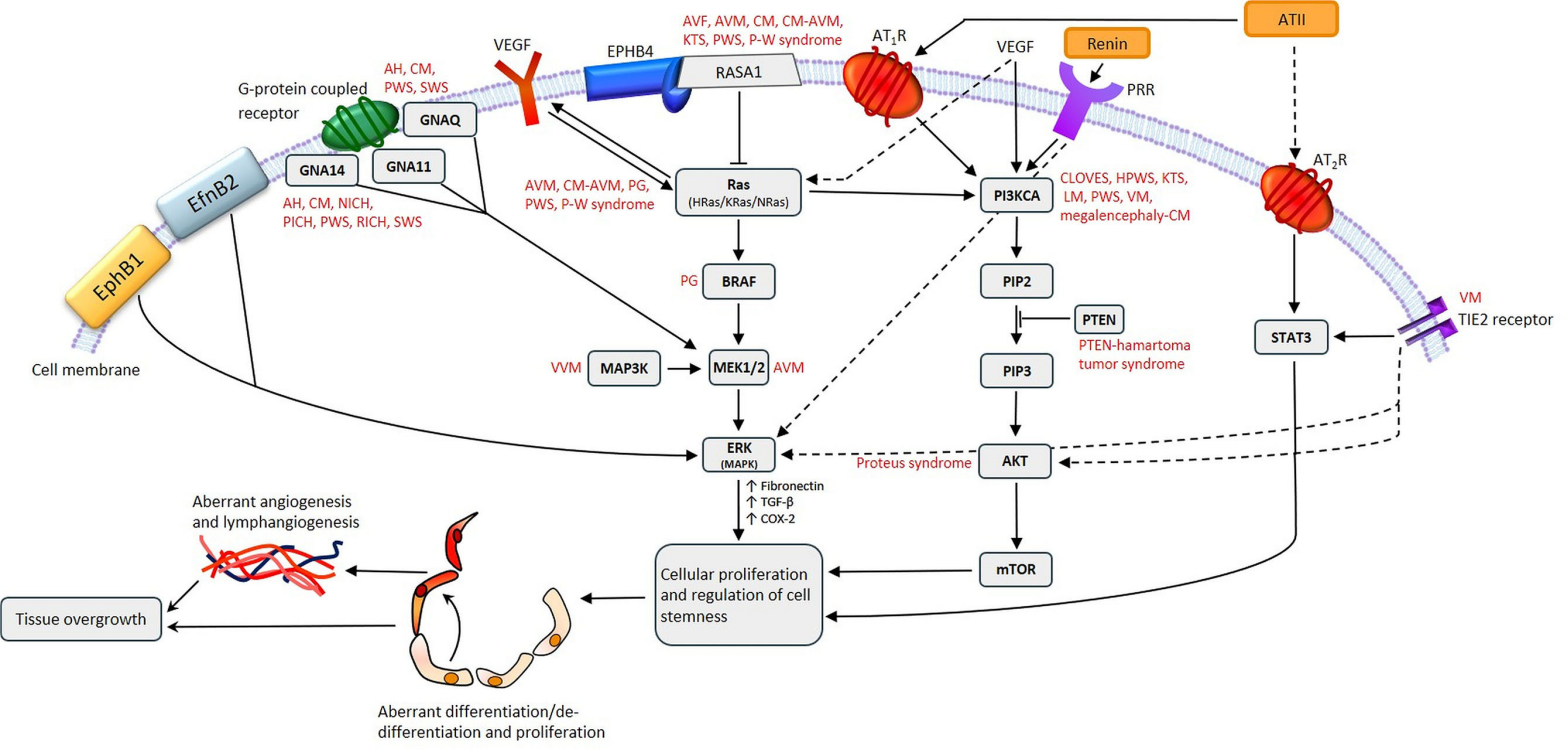
A proposed model for the role of gene mutations involving the Ras/BRAF/MEK/ERK and the PI3KCA/AKT/mTOR pathways by their interaction with different components of the renin-angiotensin system, leading to the induction and/or maintenance of cells that express stemness-associated markers in vascular anomalies. AH, anastomosing hemangioma; ATII, angiotensin II; AT_1_R, angiotensin II receptor 1; AT_2_R, angiotensin II receptor 2; PRR, pro-renin receptor; AVF, arteriovenous fistula; AVM, arterio-venous malformation; CM, capillary malformation; CM-AVM, capillary malformation—arterio-venous malformation; CM-AVM2, capillary malformation—arterio-venous malformation 2; CLOVES, congenital lipomatous overgrowth with vascular—epidermal and skeletal anomalies; HPWS, hypertrophic port-wine stain; P-W syndrome, Parkes-Weber syndrome; PWS, port-wine stain; KTS, Klippel-Trénaunay syndrome; LM, lymphatic malformation; NICH, non-involuting congenital hemangioma; PG, pyogenic granuloma; PICH, partially involuting congenital hemangioma; RICH, rapidly involuting congenital hemangioma; SWS, Sturge-Weber syndrome; VEGF, vascular endothelial growth factor; VM, venous malformation; VVM, verrucous venous malformation.

Gene mutations have been identified in some VAs, although not in IH despite intensive research (9, 13). A missense mutation in *TIE2* (Figure 2) that causes an arginine-to-tryptophan substitution at position 849 on chromosome 9, was discovered in familial venous malformation (VM) in 1996 (14). TIE2 (TEK or HYK), an endothelial-specific receptor in the tyrosine kinase sub-family (15), is critical for the regulation of vasculogenesis, vascular remodeling, endothelial cell integrity and survival, upon binding angiopoietin 1 (Ang-1) and angiopoietin 2 (Ang-2) (16). Additional germline and somatic mutations have since been identified in both familial and sporadic VM (15, 17–19).

Glomuvenous malformation, characterized by the presence of glomus cells that represent immature vascular smooth muscle cells (vSMCs) in the walls of affected venous channels, is associated with mutations in the *glomulin* gene on chromosome *1p21-22*, with 157delAAGAA being present in 48.8% of 23 affected families (20).

Fifty-four percent of VMs without detectable *TIE2* mutations are caused by somatic activating mutations in the *PIK3CA* gene (Figure 2), which encodes a catalytic subunit of the phosphatidylinositol 3-kinase (PI3K) enzyme p110α (21). Somatic mutations in *PIK3CA* are also associated with lymphatic malformation (LM) (22) (Figure 2). Disorders which feature LM, including overgrowth syndromes such as congenital lipomatous overgrowth with vascular, epidermal and skeletal anomalies (CLOVES) and Klippel-Trénaunay syndrome (KTS), are also frequently associated with somatic mutations in the *PIK3CA* gene (22) (Figure 2). Mutations affecting *PIK3CA* also cause a megalencephaly-capillary malformation (23) (Figure 2).

Somatic mutations in the *MAP2K1* gene, which encodes MEK1, are associated with arterio-venous malformation (AVM) (24) (Figure 2), and constitutively increased MEK1 activity is observed in various cancer types (25, 26).

Capillary malformation-arteriovenous malformation (CM-AVM) is an autosomal dominant vascular malformation, associated with mutations in the *RASA1* gene (Figure 2) which have also been identified in capillary malformation (CM), AVM, arterio-venous fistula, and Parkes-Weber syndrome (27) (Figure 2). Germline loss-of-function mutations in *EPHB4* are present in CM-AVM2, the second type of CM-AVM characterized by intra- and extra-cranial AVMs, multifocal CMs and telangiectasias (28).

Congenital hemangiomas are rare vascular tumors which are fully developed *in utero*. They are subcategorized into rapidly involuting congenital hemangioma (RICH), non-involuting congenital hemangioma (NICH) and partially involuting congenital hemangioma (PICH) (29, 30). RICH, NICH and PICH are associated with somatic activating mutations in *GNAQ* and *GNA11* (31) (Figure 2). *GNAQ, GNA11*, and *GNA14* mutations are present in anastomosing hemangioma (32) (Figure 2). *GNAQ* mutations have also been identified in Sturge-Weber syndrome (SWS) and CM (33, 34) (Figure 2). The Gln209 missense mutation in *GNAQ* and *GNA11* has also been demonstrated in uveal melanoma, in which it constitutively activates MAPK signaling (35).

Somatic activating mutations in *GNA14, HRas, KRas, NRas*, and *GNA11* are associated with certain vascular tumors (36) (Figure 2), and somatic mutations in *MAPK3* are associated with verrucous venous malformation (VVM) (37) (Figure 2). Furthermore, mutations in both *BRAF* and *Ras* genes have been demonstrated in sporadic and secondary pyogenic granuloma (PG) (38) (Figure 2), and mutations in *PTEN* are associated with PTEN-hamartoma tumor syndrome (39) (Figure 2). A somatic activating mutation in *AKT* has been associated with Proteus syndrome (40) (Figure 2).

The identification of a range of somatic and some germline mutations affecting different genes in different types of VAs and overgrowth syndromes have improved our understanding of these challenging conditions. The occurrence of identical mutations in multiple VAs suggests it is not the type of mutation that dictates which type of VA develops, but rather what endothelial cell lineage or stem cell type the genetic alteration occurs in during embryogenesis (41).

This review analyzes mutations in each type of VA, how they may directly influence embryonic stem cell (ESC)-like cells, and the possible interactions between these mutations and the RAS in regulating the stemness of the primitive populations within VAs (Figure 2).

### Induced Pluripotent Stem Cells

Introduction of transcription factors OCT4, SOX2, c-MYC, and KLF4 into adult mouse (42) and human (43) fibroblasts induces pluripotent stem cell (iPSC) formation. This can also be achieved with NANOG and LIN28 in place of c-MYC and KLF4 (44). The master regulators of pluripotency—OCT4, SOX2, c-MYC, and KLF4—are known as the “Yamanaka factors.” The expression of stemness-associated markers by various types of VAs (45–49) warrants further investigation including functional studies to confirm if they possess ESC properties.

### Populations of Cells Expressing Stemness-Associated Markers in Vascular Anomalies

Populations of cells expressing stemness-associated markers have been identified in different types of vascular tumors including IH (50) and PG (51), and vascular malformations including VM (46), LM (45), VVM (47), AVM (48), and port-wine stain (PWS) (49)—the most common form of CM. The observation of the proliferative nature of vascular malformations challenges the prevailing notion that they consist of quiescent non-proliferating endothelium. It is interesting to speculate that gene mutations (Figure 2) may play a key role in the induction and/or maintenance of the primitive population by influencing aberrant proliferation and differentiation, or de-differentiation of mature cells by upregulating the Yamanaka factors (43).

### The Renin-Angiotensin System and Its Bypass Loops

**Figure 3:**
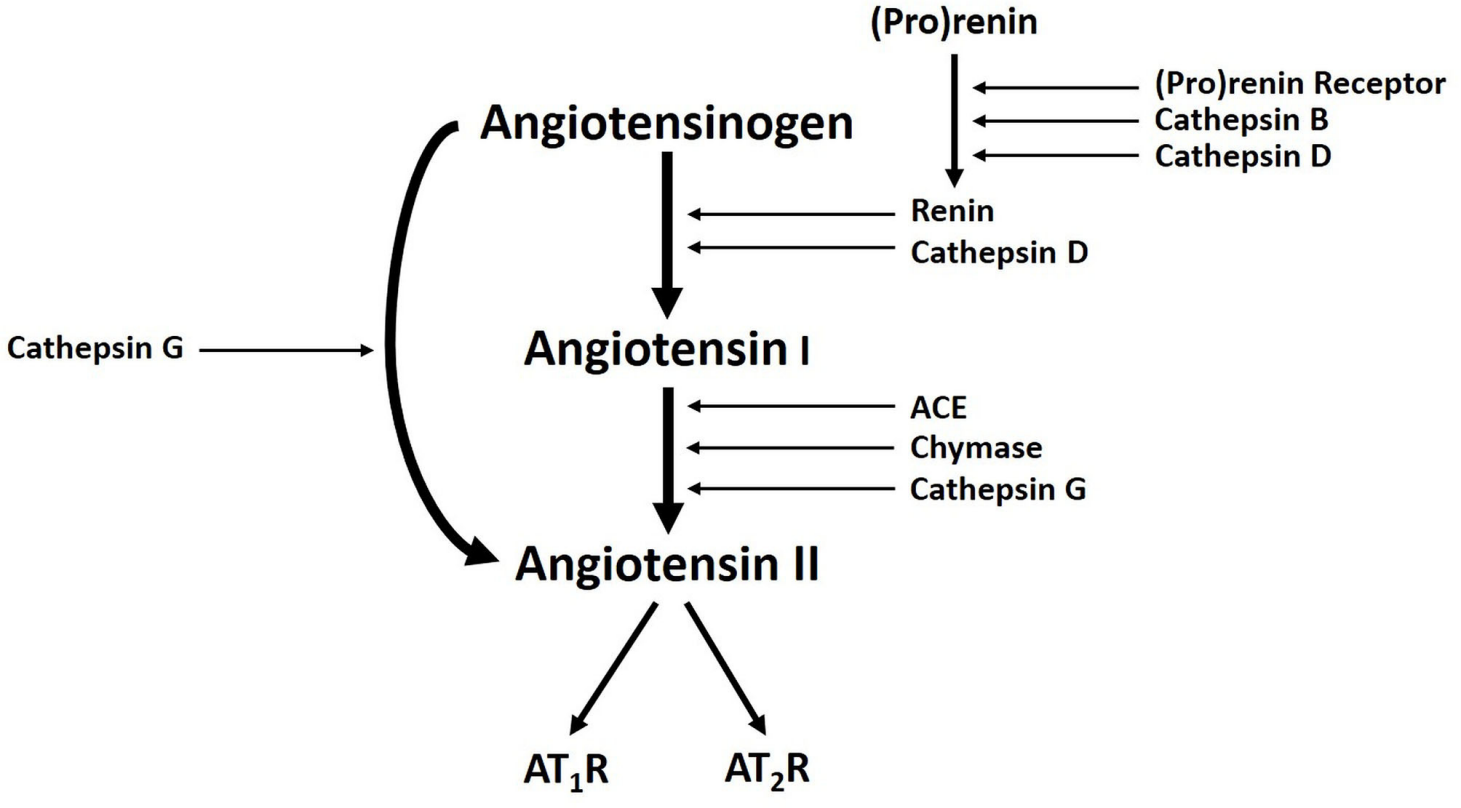
A Schema demonstrating the classical renin-angiotensin system, with cathepsins B, D, and G, acting as bypass loops. Activation of pro-renin occurs upon binding with pro-renin receptor. Renin then converts angiotensinogen into angiotensin I (ATI), which is cleaved by angiotensin-converting enzyme (ACE) to produce the active peptide angiotensin II (ATII). The actions of ATII are mediated through interactions with ATII receptor 1 (AT_1_R) and ATII receptor 2 (AT_2_R). Cathepsin B and cathepsin D contribute to renin activation. Cathepsin D and chymase mediates conversion of angiotensinogen into ATI. Cathepsin G promotes generation of ATII from ATI or directly from angiotensinogen. Reproduced with permission from Expert Rev Clin Pharmacol (6).

Populations of cells expressing stemness-associated markers within several types of VAs express components of the RAS (7, 52, 53). The classical RAS (Figure 3) is an endocrine cascade that is crucial for blood volume and blood pressure homeostasis (54). It is also a critical regulator of hematopoietic and mesenchymal stem cells (55). Renin, an aspartyl protease converted from its precursor pro-renin, cleaves angiotensinogen (AGT) to form angiotensin I (ATI) (54, 56). Angiotensin-converting enzyme (ACE) converts ATI to angiotensin II (ATII) (54, 56, 57), the physiologically active component of the cascade that exerts its physiologic effects by binding to ATII receptor 1 (AT_1_R) and ATII receptor 2 (AT_2_R) (54). There are also bypass loops of the RAS comprising enzymes such as cathepsins B, D and G and chymase (57, 58). Cathepsin B belongs to a family of cysteine proteases that converts pro-renin to renin (57). Cathepsin D, an aspartyl protease and a renin analog, cleaves AGT to form ATI (57). Cathepsin G, a serine protease, produces ATII directly from AGT or from ATI while chymase, a mast cell protease, converts ATI to ATII (57) (Figure 3). Cathepsins B, D, and G have been demonstrated in IH (59) and LM (60).

## Vascular Tumors

### Infantile Hemangioma

IH is characterized by rapid proliferation during infancy followed by slow involution over the subsequent 5–10 years (1). The identification of endothelial progenitor cells (EPCs) that co-express CD133 and CD34 in proliferating IH tissues (61) and the peripheral circulation (62) of IH patients, led to the proposal of EPCs as the origin for IH (1). However, the expression of markers associated with primitive hematopoietic cells and the transcription factors brachyury and GATA-2 by the endothelium of proliferating IH indicates an up-stream primitive mesodermal origin (63). This suggests the immature capillaries within proliferating IH are derived from a hemogenic endothelium, downstream of hemangioblasts (64), capable of undergoing mesenchymal (65, 66), neuronal (67), endothelial (67), and hematopoietic (68) differentiation. The expression of the stemness-associated markers OCT4, SSEA-4 and pSTAT3 on the endothelium of proliferating IH, implies the presence of an ESC-like population (50). STAT3 signaling plays an important role in the maintenance and stemness of cancer stem cells (69), and its activation maintains ESCs in an undifferentiated state (70). The ESC markers NANOG, SALL4 and CD133 have also been demonstrated on cells within the interstitium of proliferating IH (50).

The distinctive expression of glucose transporter-1 (GLUT-1) in IH (71) and syncytiotrophoblast membranes of the human placenta (72) and the unique co-expression of placental antigens FCγRIII, Lewis Y antigen and merosin, and type 3-iodothyronine deiodinase on IH, led to the speculation of a placental embolic origin of IH (1, 73–75). The presence of human chorionic gonadotrophin, human placental lactogen, but not human leucocyte antigen-G or cytokeratin 7, on proliferating endothelium of IH, suggests a placental chorionic villous mesenchymal core origin of IH (76).

The endothelium of the microvessels within proliferating IH expresses embryonic hemoglobin ζ (HBZ) and erythropoietin receptor (EPOR) (68). Erythropoietin is a crucial growth factor for the proliferation, differentiation and survival of erythroid progenitor cells (77). EPOR is found with HBZ in extra-embryonic yolk sac blood islands (77) – the location of primitive hematopoiesis during the first trimester of pregnancy (78, 79). Cell culture of IH explants *in vitro* form enucleated erythrocytes which express the erythrocyte-specific marker glycophorin A (68). These findings suggest that the endothelium of IH is a functional hemogenic endothelium which may be a site of primitive extra-medullary erythropoiesis (68).

The endothelium of proliferating IH also expresses the neural crest markers p75 (a neurotrophin receptor), SOX9 and SOX10 (63), NG2 and nestin (80), suggesting a primitive mesoderm with a neural crest phenotype (63). The segmental distribution of the IH seen in PHACES syndrome (63, 81), is reminiscent of the migratory paths of neural crest cells during embryogenesis (82).

Accelerated involution of proliferating IH induced by propranolol (83), acebutolol (84), other β-blockers (85, 86) and captopril (5), suggests a role for the RAS in the pathogenesis for IH (1, 6). The CD34+ hemogenic endothelium of proliferating IH expresses components of the RAS: ACE and AT_2_R, but not AT_1_R (7). Pro-renin receptor (PRR), another component of the RAS, is expressed on both endothelial and non-endothelial cell populations of IH. Renin has been demonstrated to promote proliferation of the endothelial cell population that may be blocked via inhibition of the canonical Wnt signaling pathway, using the Wnt receptor blocker dickkopf-1 (87). These findings suggest there are interactions between renin, PRR and Wnt signaling (88). This is unsurprising given PRR is a part of the Wnt/frizzled receptor complex (89).

Explants of proliferating IH form blast-like structures in media upon administration of ATII in a dose-dependent manner (90), which could explain the accelerated involution of proliferating IH induced by propranolol (7). This is supported by the observation that serum renin levels are five times higher in the first 3 months of life and remain three times higher at 3–12 months old, when compared to adults. Serum renin levels then taper to normal adult levels from 8 years of age (91). This gradual decline in serum renin levels reflects the programmed biologic behavior of IH (1, 92). Patients with proliferating IH treated with surgery or propranolol have significantly decreased plasma renin levels (82), whereas surgery and captopril administration significantly reduce the mean levels of ATII. However, none of these interventions significantly affect the mean serum levels of ACE (93). The significant changes in renin and mean levels of ATII following these interventions further evidences the crucial role of the RAS.

The observation that the angiotensin peptides significantly influence cellular proliferation of IH underscores a central role of the RAS in the pathobiology of IH. While explants of IH treated with an ACE or AT_2_R antagonist demonstrate a significant reduction in the nuclear expression of Ki67, a marker of cellular proliferation, the addition of an AT_2_R agonist increases the number of Ki67+ cells (90). ACE is expressed on the endothelium of IH (7). The ACE inhibitor ramipril blocked AT1-induced proliferation on IH explant *in vitro* (90). This shows that ATI and ATII promote cellular proliferation in proliferating IH through AT_2_R signaling (90). This suggests that conversion of ATI to the vasoactive peptide ATII occurs in a paracrine fashion within IH, i.e., the presence of a local (paracrine) RAS (7).

Phosphorylated forms of STAT1, STAT3, and STAT5 have been demonstrated in proliferating IH (94). This is significant as the STAT family consists of intracellular signaling molecules with multiple influences on stem cell populations, particularly hematopoietic stem cells (95). They translocate to the nucleus to influence gene expression upon latent STATs undergoing Janus-kinase (JAK)-mediated phosphorylation and subsequent dimerization (96). STAT activation decreases markedly and is not expressed by some cells in involuted IH. This decrease may reflect depleting stem cell numbers present within involuting IH as it transitions to a fibro-fatty residuum, and suggests that reduced STAT expression decreases stem cell maintenance (94). Binding of ATII to AT_2_R increases activation of STAT3 (94). This may explain the widespread activation of STAT3 in proliferating IH, underscoring the role of the RAS in spontaneous involution and accelerated involution of IH induced by RAS modulators (94). Functional work is needed to determine if accelerated involution induced by RAS-modulating therapies is due to decreased STAT3 signaling which has been thought to cause the loss of stem cell maintenance (94).

Propranolol is administered as a 1:1 racemic mixture of both R(+) and S(–) stereoisomers (97). Recent evidence demonstrates that the R stereoisomer of propranolol which is inactive against the β-adrenergic receptor, inhibits the growth of bEnd.3 hemangioma cells *in vivo* (98). R-propranolol is a small molecule inhibitor of the transcription factor SOX18 (97). Mutations in *SOX18* cause hypotrichosis-lymphedema-telangiectasia and renal syndrome (HLTRS), which feature vascular and lymphatic defects (99). Long-term use of propranolol is effective for treating HLTRS (100), providing further evidence propranolol does not only act via β-adrenergic pathways (97). Induced differentiation of hemangioma-derived stem cells (HemSC) into hemangioma endothelial cells with VEGF-B, increases SOX18 expression. Treatment of HemSC with propranolol significantly reduces VEGF-B-induced upregulation of all endothelial markers investigated to a magnitude similar to the SOX18 inhibitor Sm4 (97). Moreover, when repeated with purified R(+) and S(−) enantiomers, only R-propranolol reproduces the endothelial cell marker inhibition seen with racemic propranolol. This indicates there may be a SOX18-dependent pathway, independent of β-adrenergic blockade, influencing HemSC differentiation (97). This suggests the possibility of treating IH independent of β-adrenergic blockade, thereby avoiding side effects of β-blocker therapy.

These findings have led to the proposal that IH originates from aberrant proliferation and differentiation of primitive mesoderm-derived hemogenic endothelium (63) with a neural crest phenotype (101) and a placental chorionic villous mesenchymal core origin (76), regulated by the RAS (3). Further functional studies are needed to fully determine the role of the RAS in the programmed biologic behavior of IH, and its accelerated involution induced by β-blockers and ACE inhibitors (3, 6, 102).

### Pyogenic Granuloma

Populations of cells that express stemness-associated markers have been identified in the endothelium of the microvessels and the interstitium of PG, another common type of benign vascular tumor (51). The primitive subpopulation on the endothelium expresses the ESC markers OCT4, SOX2, NANOG, and pSTAT3, whereas a subpopulation within the interstitium expresses SOX2, NANOG, and pSTAT3 (51). These subpopulations in PG also express components of the RAS (52). The expression of ACE on the endothelium of the microvessels suggests paracrine conversion of ATI to ATII, which may impact cellular proliferation and angiogenesis within PG (54, 103). It has been proposed that the presence of AT_1_R on the microvessels within PG may contribute to immature microvessel formation by influencing the ESC-like population to differentiate down an endothelial phenotype (52, 104). If cells expressing stemness-associated markers preferentially differentiate toward an endothelial phenotype under the influence of the RAS, this could be targeted by RAS modulators (52).

Somatic gain of function mutations of *HRas* (Figure 2) have been identified in PG (105) with *Ras* signaling being associated with microvessel proliferation and angiogenesis (105). Further, *KRas* activation increases VEGF synthesis (106) (Figure 2), which at increased levels stimulates mobilization of hematopoietic stem cells and hematopoietic progenitor cells, and a cell population capable of rapid endothelial colony formation. This suggests an immature endothelial cell phenotype within PG (107). These mobilized hematopoietic stem cells and hematopoietic progenitor cells are comparable to the KDR+/CD34+/AC133+ cells seen in G-CSF mobilized blood, bone marrow and umbilical cord (108). It is interesting to speculate whether elevated VEGF expression caused by *Ras* mutations leads to increased levels of circulating precursor cells, and whether these are a source of the previously identified primitive cells within PG.

## Vascular Malformations

### Venous Malformation

Venous malformation (VM) represents the most common vascular malformation (109), whereby affected veins consist of a thin endothelial cell lining surrounded by sparsely and erratically distributed vSMCs which are sometimes absent (21). This, and the disordered extracellular matrix deposition, lead to dysfunctional basement membrane development and irregular endothelial cell monolayer arrangement, forming irregularly shaped ectatic vessels prone to thrombosis (21, 110). There are four subtypes of VM: sporadic VM, cutaneo-mucosal VM (CMVM), multifocal VM, and blue rubber bleb nevus syndrome (BRBN) (9).

Populations of cells that express the stemness-associated markers OCT4, NANOG, SOX2, SALL4, pSTAT3, and CD44 have been demonstrated within the endothelium of the lesional vessels and the interstitium in both subcutaneous VM (SCVM) and intramuscular VM (IMVM) (46). The endothelial sub-population which expresses OCT4, sitting atop the stem cell hierarchy, may differentiate and give rise to the interstitial subpopulation (46). *c-kit*, a stem cell growth factor receptor, has also been identified in the smaller lesional vessels in BRBN (111). This suggests that the growth of VM is associated with the *c-kit* signaling axis (111).

ACE and PRR have been demonstrated on the endothelium of the lesional vessels in VM (112). PRR binds pro-renin which facilitates increased local conversion of AGT to ATI (112) (Figure 3). PRR also functions in downstream signaling through the MAP kinases ERK1 and ERK2 to upregulate TGF-β1, PAI1, collagens, fibronectin (87, 113, 114) and cyclooxygenase-2 (115) (Figure 2). Some of these downstream effectors, particularly TGF-β1 and fibronectin, play a role in cellular growth, proliferation and differentiation (116) (Figure 2). These functions highlight the potential role of PRR in VM pathobiology (112).

The proangiogenic effects of ATII occur through its interactions with AT_1_R (117) (Figure 2). The endothelium of SCVM and IMVM expresses AT_1_R, and the endothelium of smaller vessels stains more strongly than that of dilated vessels by immunohistochemical staining (112). ATII has proangiogenic effects, when acting through AT_1_R, providing a possible explanation for the increased density of abnormal venous channels in VM (112). Further, AT_2_R is expressed on both endothelial and non-endothelial cells (112) and signaling through this receptor stimulates blast cell proliferation and their differentiation into either endothelial or hematopoietic progenitor cells (104).

Germline or sporadic gain of function mutations in *TIE2* have been identified in ~60% of VM cases (18, 118) (Figure 2). *PIK3CA* mutations have also been demonstrated in about 25% of VM cases (119) (Figure 2). Although co-occurring mutations in *TIE2* and *PIK3CA* are rare, they both result in PIK3 signaling and subsequent AKT activation (41) (Figure 2). TIE2, through its interaction with its ligand Ang-1, is involved in the recruitment of both pericytes and vSMCs (120). Ang-1, a glycoprotein, is a member of the angiopoietin family of growth factors and promotes vascular quiescence and structural integrity (121). Given the functional role of Ang-1 in vascular quiescence, we speculate that *TIE2* dysregulation plays a role in the loss of quiescence, hence challenging the notion that VM is not a proliferative lesion. Dysfunction in TIE2 precipitates activation of STATs (120) and activation of the AKT phosphorylation pathway (122) (Figure 2). As a result, the affected vessels lack vSMCs, resulting in ‘blow-out' of the affected channels in VM (123). STAT1 is involved in inhibition of endothelial cell growth, while STAT3 is involved in angiogenesis and endothelial activation (124). Both are induced by the *TIE2* mutant variant R849W; however, STAT3 is implicated in the pathogenesis of many cancer types (124). STATs have also been identified in proliferating IH and their role in stem cell maintenance has been suggested (94). Given subpopulations of cells that express stemness-associated markers have been identified in VM, it would be interesting to investigate whether the stem cell maintenance function of STATs also play a role in the pathogenesis of VM. Additionally, components of the RAS have been implicated in STAT expression, as the binding of ATII to AT_2_R activates STAT3 (94) (Figure 2). This provides some evidence for the interaction between genetic mutations, including those of *TIE2*, and the RAS, suggesting they act synergistically on STAT pathways to influence stemness. R849W, the most common germline mutation affecting *TIE2*, causes an arginine-to-tryptophan substitution at position 849 (19). It causes partial hyper-phosphorylation of TIE2 and requires a second somatic mutation for phenotypic penetration (19). *TIE2* mutations have been implicated in ~60% of sporadic VM cases (125). Sixty percent of these have been attributed to L914F, a somatic mutation that causes a phenylalanine-to-leucine substitution at position 914 resulting in dysfunctional vascular development and cell migration (126). Approximately half of the remaining 40% of sporadic VM cases are attributed to mutations in *PIK3CA* (Figure 2), and the resultant PI3K signaling dysfunction (21), leading to upregulation of the protein kinase AKT (21). Somatic mutations in this gene have been identified in overgrowth syndromes including VM, and many cancer types (21). Increased AKT signaling has been suggested to promote endothelial cell survival and proliferation in vSMC-deficient malformed veins typically found in VM (119, 126–128).

There is increasing evidence demonstrating interactions between gene mutations and the RAS in VM (129, 130). Cheng et al. (131) demonstrate the role of renin in activating the PI3K signaling pathway (Figure 2). ATII, acting downstream of renin also activates the PI3K pathway by binding to AT_1_R, as well as stimulating G protein subunit q (131) (Figure 2). Further research is needed to determine whether the same interaction occurs between RAS and the PI3K signaling pathway in VM.

The expression of components of the RAS by ESC-like subpopulations within VM may underscore the observed effect of propranolol, a RAS inhibitor (132), and celecoxib, a COX-2 inhibitor which causes upregulation of PRR (133), on a patient with troublesome VM (134). The clinical response to β-blockers has been hypothesized to be caused by a reduction in plasma renin levels (135), while COX-2 inhibitors potentially act to prevent ATII-induced expression of PRR (133). Additionally, pharmaceuticals targeting the PI3K pathway, such as sirolimus (an mTOR inhibitor), have proved effective in refractive cases of VM (21). Lesion growth and vascular volume of VM have been shown to decrease in mice following sirolimus treatment, whilst radiological lesion size, bleeding, pain and esthetic impairment are reduced following administration of sirolimus in a small number of patients (136, 137). Sirolimus binds to and interferes with the intracellular receptor on mTOR, FKBP12 (138). Given mTOR's role in cellular growth and proliferation, the response of VM to sirolimus challenges the notion that vascular malformations contain a quiescent endothelium (10).

### Verrucous Venous Malformation

VVM, formerly known as *verrucous hemangioma* (9), is a subtype of VM that presents clinically as a vascular malformation but with histopathological characteristics of a vascular tumor (139). A significant proportion of these non-hereditary, congenital lesions contain a somatic missense mutation in *MAP3K* (37). These lesions are present at birth and grow commensurately throughout infancy (140). They eventually form friable, dark black-blue, hyperkeratotic plaques which bleed easily with minor trauma (140). The endothelium of VVM expresses GLUT-1, the transcription factor OCT4 and brachyury—a marker of primitive mesoderm, and also ACE—the central component of the RAS (9).

### Lymphatic Malformation

LM affects 1:5,000 births (141) and may be identified prenatally, at birth, or during childhood (142). LMs fall under three of the four groups of vascular malformations: simple malformations, combined malformations, and malformations of major named vessels (9). LMs are further categorized into microcystic or macrocystic, or mixed subtypes (9). LM commonly affects the cervicofacial and axillary regions (9). LMs consist of thin-walled dilated channels or cysts with a lymphatic morphology lined by flat endothelial cells and few pericytes (9) surrounded by stroma (141). Somatic activating mutations in *PIK3CA* have been identified in both isolated LMs and those associated with several overgrowth syndromes (22) (Figure 2).

A cell population that expresses the stemness-associated markers OCT4, NANOG, SOX2, KLF4, and c-MYC on the D2-40+ endothelium of the lesion vessels, and c-MYC and SOX2 within the fibrous stroma have been demonstrated in microcystic LM (45). Further, endothelial cells lining aberrant lymphatic vessels within LM and in surrounding stroma express CD133 (143). Isolated CD133+ cells express NANOG and OCT4, and markers found on circulating endothelial precursor cells such as CD90, CD146, *c-kit* and VEGFR2 (143). These CD133+ cells also express the lymphatic endothelial markers D2-40 and VEGFR3 (45, 143). CD133 is expressed by hematopoietic stem cells and EPCs, but not ESCs (45, 144). These CD133+ progenitor cells are multipotent, and are able to differentiate into bone, smooth muscle, fat, and lymphatic endothelial cells *in vitro* (143). *In vivo*, CD133+ LM cells differentiate into lymphatic endothelial cells giving rise to dilated lymphatic channels characteristic of human LMs (143). Further, LM-derived cells demonstrate increased proliferation, migration, and resistance to apoptosis, *in vitro* (145). The presence of progenitor cells capable of multipotent differentiation supports a potential role for aberrant ESC-like cells in the pathogenesis of LM. We speculate that these aberrant progenitor cells are downstream to a more primitive ESC-like population, which may include a cell population that expresses stemness-associated markers in microcystic LM (45).

### Arterio-Venous Malformation

AVM consists of a tangle of arteries and veins providing direct connections between high-flow and low-flow vessels (146) forming the central *nidus*, bypassing the normal capillary network (146).

Although the pathogenesis of AVM remains unclear, there is evidence showing that AVM may arise from errors in angiogenesis during 4th−6th week of gestation that leads to aberrant vessel remodeling (147–149). It has been proposed that a gene mutation causes aberrant signaling in pathways, such as the PI3K/AKT and mTOR pathways, which are associated with angiogenesis and vascular development (24) (Figure 2). Ras (not to be confused with the RAS), a series of GTPases and a variety of tyrosine kinase receptors, modulate the PI3K/AKT pathway to enable generation of lipid products (24, 150, 151). These lipid products contribute to normal vascular development and angiogenesis, and play a role in cellular survival, proliferation, and cytoskeletal re-organization (24, 150, 151). The mTOR complex lies downstream of PI3K signaling, and collectively the PI3K/AKT/mTOR pathway is a major regulator of cell survival (150–152) (Figure 2). Ras proteins also play a role in the activation of the MAPK cascade (24). The role of MAPK is extensive in regulating gene expression and cell proliferation, differentiation and survival (24, 153). Several studies have revealed pathogenic variants in the Ras/MAPK pathways in patients with AVM (24, 154, 155) (Figure 2). Further, somatic mutations have been identified in *MAP2K1*, which encodes for MEK1, in AVM (24) (Figure 2). MEK inhibitors are in clinical use for the treatment of several cancer types (156, 157), and result in clinical improvement in patients with central conducting lymphatic anomalies with recurrent *ARAF* mutations (158). These mutations in MAPK's negative regulatory domain (24) are similar to those seen in malignant melanoma (159) and lung cancer (160, 161). The potential use of this targeted therapy in AVM warrants investigation.

Populations of cells that express the stemness-associated markers OCT4, SOX2, KLF4, and c-MYC have been identified on the endothelium and media of the lesional vessels, as well as cells within the stroma of the *nidus* of AVM (48). Zheng et al. (162) propose that the PI3K/AKT pathway lies downstream of ATII receptors in ESCs, as ATII upregulates phospho-Akt, and show that ATII promotes differentiation of mouse ESCs to form SMCs through activation of ATR_1_ (162). We propose that an interaction may exist between gene mutations and the cells that express stemness-associated markers that may influence the pathophysiology of AVM (Figure 2), similar to VM. The precise relationship between the ESC-like population and the RAS warrants further investigation including the expression of components of RAS, particularly ATII, in AVM. Further, over-activation of the MAPK pathway affects SOX2 in pre-gestational models, increasing cellular proliferation and impairing terminal differentiation (163). Increased clonogenic capacity of SOX2 may be prolonged, in part, by the MAPK pathway (163) and errors causing dysregulation in this pathway, including the aforementioned pathogenic variants, contribute to the inexorable nature of AVM.

### Port-Wine Stain

PWS, or *nevus flammeus*, is the most common type of CM (164). It affects about 26 million people globally (164, 165) and is characterized by an increased number of dilated venular-capillary vessels within the dermis (9). Fifteen to Twenty percent of PWS affecting the V1 dermatome of the trigeminal nerve are associated with SWS (165). PWS is also part of KTS and Parkes-Weber syndrome (166).

The standard treatment of PWS is pulsed dye laser requiring multiple treatment sessions (167), with persistence remaining a challenge (168). Surgical de-bulking is used for hypertrophic PWS (HPWS) with modest results (169).

There are two main hypotheses for the pathogenesis of PWS: denervation and gene mutations (165). Most facial PWS show trigeminal nerve dermatomal distribution, along V2 (32%), combined V1-V2 (41%), combined V2-V3 (5%), and involvement of V1-3 (10%) (170). Microvessels within the middle and deep dermis of PWS lack normal innervation (171, 172). Also, the nerve fibers in PWS do not respond to epinephrine administration *in vitro*, implying a defective sympathetic basal tone as a cause for the vessel dilation seen in PWS (165, 173). “Denervation” leads to decreased basal sympathetic tone, and the loss or decrease of neuronal factors has been postulated to cause PWS (165). Alternatively, it is interesting to speculate that aberrant neural differentiation from sympathetic neuronal progenitor cells may have caused deficient innervation and sympathetic tone.

**Figure 4:**
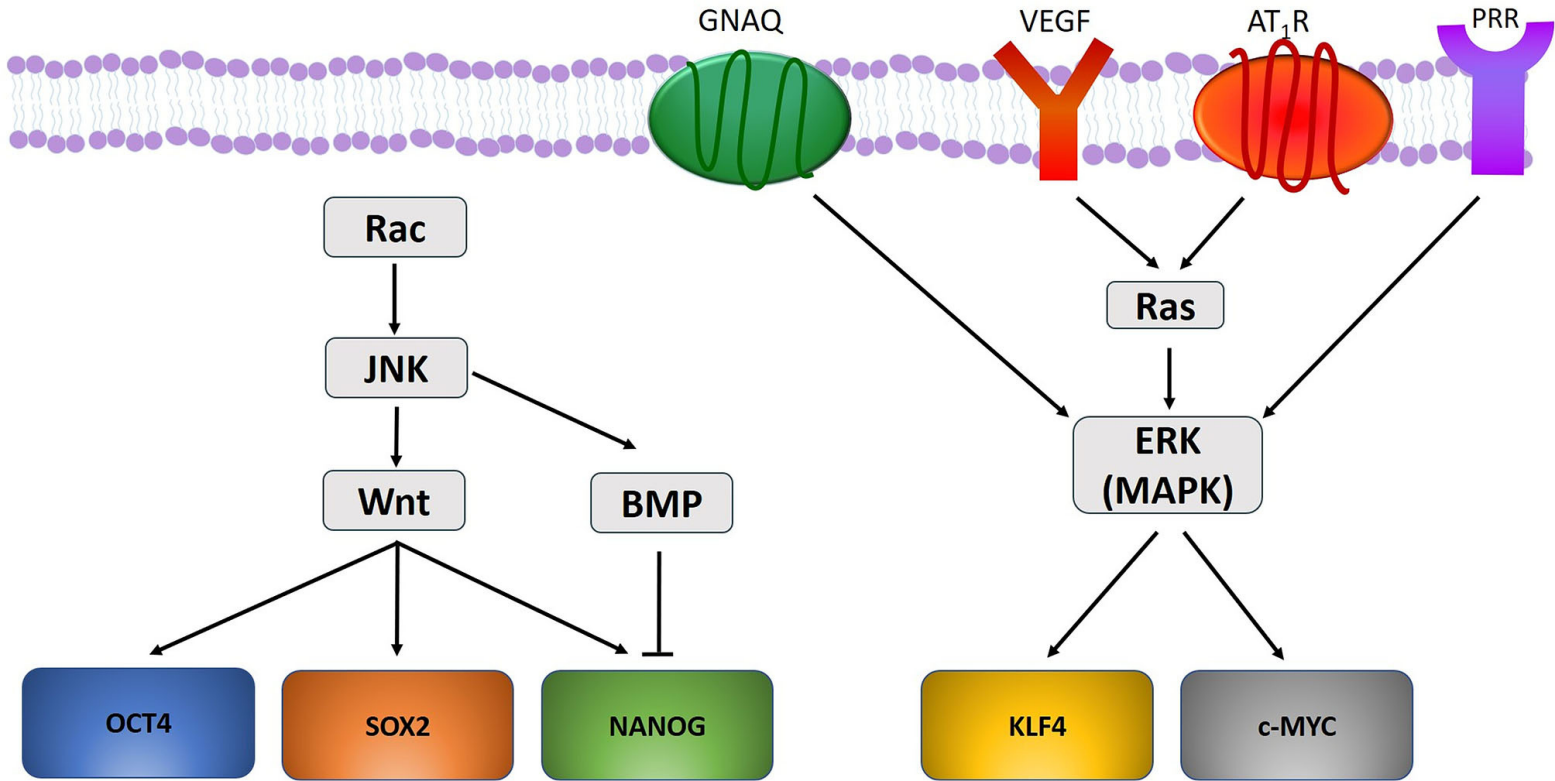
A proposed model portraying how gene mutations identified within vascular anomalies affect the expression of the stemness-associated markers OCT4, SOX2, NANOG, KLF4, and c-MYC. OCT4, SOX2, and NANOG are upregulated through Wnt signaling, and NANOG is down regulated through BMP-signaling. KLF4 and c-MYC are upregulated by ERK activation under the influence of GNAQ, VEGF, and RAS signaling via activation of angiotensin II receptor 1 (AT_1_R) and pro-renin receptor (PRR).

There may be a link between the gene mutations and the ESC-like population in PWS (49). Mutations in *GNAQ* (R183Q) (33) and *PI3K* (G1049N) (165, 174) have been demonstrated in PWS, and aberrant MAPK and/or PI3K signaling during embryonic development may contribute to PWS formation and SWS (165) (Figure 2). Further, a somatic mutation in *GNAQ* activates ERK via MEK, which may contribute to PWS development (33). Like ERK, c-Jun N-terminal kinases (JNK) are activated in PWS, which is also associated with the development of PWS (175). Activation of ERK via Ras signaling induces phosphorylation of KLF4 (176) and c-MYC (177). Activation of JNK via the Rac pathway induces expression of Wnt and BMP-signaling, both of which are critical in the self-renewal capability of ESCs. Wnt signaling upregulates the transcription factors OCT4, SOX2, and NANOG, whilst BMP limits NANOG activity to cause differentiation of ESCs (178) (Figure 4). VEGF, which is overexpressed in PWS (179), significantly increases Ras signaling which activates ERK, thus increasing the expression of stem cell markers which may bolster and sustain the resident ESC-like population in PWS (49) (Figure 4).

Mutations in *EphB4, RASA1, TIE2*, and co-expression of Eph receptor B1 (EphB1) and ephrin B2 (EfnB2) lead to MAPK activation (165) (Figure 2). EphB1 forward signaling and EfnB2 reverse signaling activate MAPK pathways (165). Nguyen et al. (165) propose that EphB1 and EfnB2 co-expression lead to MAPK activation. A mutation in *PI3K* causes activation of the AKT/mTOR pathway, as does the overexpression of VEGF-A and VEGFR2 (165). Defective signaling in the MAPK and PI3K pathways leads to abnormal cellular proliferation, survival, migration, cytoskeletal arrangement and vascular permeability (165). These changes ultimately lead to the development of PWS/SWS (165).

*RASA1* mutations have been demonstrated in familial AVM, SWS, KTS and Parkes-Weber syndrome (166, 180, 181) (Figure 2), suggesting that germline mutations in *RASA1* are associated with familial susceptibility to these VAs (165). A somatic mutation in *PIKC3A* (G1049N) has been demonstrated in HPWS (174) (Figure 2). *PIKC3A* (G1049N) mutation increases endothelial cell proliferation *in vitro* (182), which may occur in PWS (165, 182). Somatic mutations in *SMARCA4, EPHA3, KRas, NRas, MAP2K1*, and *PDGFR-*β have also been identified in PWS (165, 174) and they may act as “second hit” mutations for germline mutations such as *RASA1*, to create the PWS phenotype (165).

Populations of cells expressing the stemness-associated markers OCT4, SOX2, KLF4 and c-MYC but not NANOG, have been demonstrated in HPWS (49). It is proposed that the absence of NANOG in PWS supports the potential role of JNK in PWS, since JNK activates BMP, a NANOG suppressor (49). The presence of primitive populations within PWS is also supported by evidence demonstrating that endothelial cells within PWS co-express CD133 and CD166, markers shown to also be co-expressed by cells with EPC-like function (183). These cells displaying an EPC phenotype also express both the venous and arterial markers EphB1 and EfnB2, respectively. Co-expression of these markers on normal human dermal endothelial cells induces morphologic characteristics of PWS *in vitro* (183). It is thought that co-expression of EphB1 and EfnB2 prevents normal differentiation of arterioles and venules from the primitive capillary plexus, resulting in a pre-determined fate to form venule-like channels (183). PWS may result from these differentiation-impaired EPCs that co-express EphB1 and EfnB2, to develop into venule-like structures that progressively dilate (165). Further, cells from connective tissues, hair follicles and glands in PWS also have a *GNAQ* mutation, suggesting pluripotent cells may give rise to multiple lineages in PWS (184). The presence of *GNAQ* mutation in cells within different tissue types suggests these cells originate from an upstream ESC-like subpopulation recently identified (49).

## mTOR Inhibition in Vascular Malformations

Sirolimus is increasingly used for complex vascular anomalies. mTOR is a conserved serine/threonine protein kinase that regulates cellular proliferation, cell growth, motility and survival, protein synthesis, and transcription (185, 186). It consists of two functionally distinct complexes—mTORC1 and mTORC2, each processing different substrates (187). mTOR coordinates normal cellular growth through regulation of ribosome creation and protein synthesis by integrating signals from the PI3K/AKT pathway. Increased activation of these pathways has been demonstrated in overgrowth syndromes which include VAs (150).

Sirolimus was first administered to an infant with kaposiform hemangioendothelioma (KHE) with Kasabach-Merritt phenomenon (KMP) refractory to other therapies (8), based on the high lymphatic component within this tumor, and that the PI3K/AKT/mTOR pathway is activated in angiogenesis and lymphangiogenesis. Sirolimus has also been used to treat lymphangioleiomyomatosis, tuberous sclerosis and neurofibromatosis (8). Subsequent discovery of somatic mutations in the PI3K/mTOR (*PIK3CA*) pathway (188), and both germline and somatic mutations in related pathways in VAs, provide further rationale for inhibiting mTOR in the treatment of isolated complex VAs not associated with overgrowth syndromes (126, 189, 190).

Since its initial use in KHE, several case reports, retrospective case series and clinical trials have shown positive results with sirolimus (137, 150, 191, 192). A recent systematic review (193) analyzing two randomized controlled trials, two non-randomized prospective trials, and 69 retrospective case reports and case series, involving a total of 373 patients, provides evidence of the efficacy of sirolimus in VAs. It shows oral sirolimus is highly effective in treating vascular tumors associated with KMP, with 95.5% of patients showing clinical improvement and 93% showing normalization of coagulopathy. Reduced size of VM is observed in 89.9% of patients, and clinical improvement of LM in 94.9% of patients, but improvements have not been observed in AVM (193).

Sirolimus is associated with potential side effects, which can be broad and multi-system, most commonly oral mucositis, dyslipidemia, leukopenia, gastrointestinal symptoms, paronychia and eczema (193). Serious infection and increased risk of lymphoproliferative disease have also been reported (193, 194).

mTOR pathways regulate somatic cell reprogramming, with short-course treatment using sirolimus showing enhanced somatic cell reprogramming, whereas longer-course treatment and mTOR-knockout decreases reprogramming (195). mTOR activation in human somatic cells with ectopic expression of OCT4, SOX2, KLF4, and c-MYC, significantly increases production of iPSCs (185). mTOR inhibition induces a paused pluripotent state in mouse blastocysts which have globally suppressed transcription, but maintain their gene expression signature and pluripotency. mTOR therefore regulates developmental timing at the peri-implantation stage (196). Based on the mechanism of action of sirolimus and its association with improved clinical outcomes in VAs, it is interesting to speculate whether mTOR also affects reprogramming and proliferation of the cells that express stemness-associated markers within VAs. This also raises the possibility that sirolimus may reduce or inhibit stemness conferred by the presence of these transcription factors in VAs in a manner dependent on the dose and duration of treatment.

## Targeted Therapies for Vascular Anomalies

The discovery of germline and somatic mutations and associated variants in VAs and overgrowth syndromes (Table 1), underscores an exciting prospect of targeted therapy using new and existing agents to target these mutations and related pathways for these challenging conditions (197).

**Table 1:** Expression and location of components of the renin-angiotensin system, and gene mutations in vascular anomalies.

	**Component of the RAS expressed**	**Location of RAS components**	**Gene mutations**
Infantile hemangioma	ACE, AT_2_R, PRR	Endothelium of microvessels: PRR, ACE, AT_2_RNon-endothelial cells: PRR	None identified
Pyogenic granuloma	ACE, AT_1_R, AT_2_R, PRR	Endothelium of microvessels: PRR, ACE, AT_2_R, AT_1_RPerivascular cells: PRR, AT_1_R	*BRAF*^δ^*, Ras*^δ^
Venous malformation	ACE, PRR, AT_1_R, AT_2_R	Endothelium: PRR, ACE, AT_1_R, AT_2_RPerivascular cells: AT_2_R	*TIE2*^ζ^*, PIK3CA*^ζ^
Verrucous venous malformation	Not investigated	Not investigated	*MAP3K3*^δ^
Lymphatic malformation	Not investigated	Not investigated	*PIK3CA*^ζ^
Arterio-venous malformation	Not investigated	Not investigated	*MAP2K1*^δ^, *RASA1*^δ^
Port-wine stain	Not investigated	Not investigated	*GNAQ*^δ^*, GNA11*^δ^
Glomuvenous malformation	Not investigated	Not investigated	*Glomulin*
Capillary malformation	Not investigated	Not investigated	*GNAQ*^δ^*, GNA14*^δ^
Anastomosing hemangioma	Not investigated	Not investigated	*GNAQ*^δ^*, GNA11*^δ^*, GNA14*^δ^
RICH, NICH, PICH	Not investigated	Not investigated	*GNAQ*^δ^, *GNA11*^δ^
CM-AVM	Not investigated	Not investigated	*RASA1*^δ^
Megalencephaly-CM	Not investigated	Not investigated	*PIK3CA*^ζ^*, AKT3*^ζ^

ACE, angiotensin-converting enzyme; AT_1_R, angiotensin II receptor 1; AT_2_R, angiotensin II receptor 2; PRR, pro-renin receptor; RAS, renin-angiotensin system; CM-AVM, capillary malformation—arteriovenous malformation; CM, capillary malformation; RICH, rapidly involuting congenital hemangioma; NICH, non-involuting congenital hemangioma; PICH, partially involuting congenital hemangioma;

ζ PI3K/AKT/mTOR pathway affected;

δ *Ras/RAF/MEK/ERK pathway affected*.

Gene mutations affecting the PI3K/AKT/mTOR and the Ras/RAF/MEK/ERK pathways are the two main pathways implicated in the pathogenesis of VAs. They control cellular growth, differentiation and transcription and they interact at multiple levels (154) (Figure 2).

A recent report on the treatment of patients with CLOVES (part of the *PIK3CA*-related overgrowth syndromes) using the PIK3CA inhibitor BYL719, shows promising results. This targeted therapy improved symptoms in all patients, with a decrease in the size of the intractable VAs, improvement of congestive heart failure and cardiac hemihypertrophy, and scoliosis (198). No significant side effects were reported (138, 193). Several other PI3K inhibitors are being clinically investigated for various cancer types (199). In a pre-clinical model of AVM using BRAF-mutated zebrafish, treatment with the orally active inhibitor of mutated BRAF, vemurafenib, used clinically to treat BRAF-mutated metastatic melanoma, restores normal blood flow (154).

Targeting mutations in *MEK1/2 (MAP2K1)* in VAs with MEK inhibitors has been proposed. MEK inhibitors are currently used to target the MAPK2K1/ERK pathway in multiple cancer types, including advanced soft tissue sarcoma and metastatic melanoma (200, 201). Blockade of AKT and PI3K signaling may inhibit metastatic gastric cancer (202). Repurposing cancer therapeutics that target mutations affecting components of these key pathways in VAs warrants further investigation.

Clinical trials are underway investigating miransertib (ARQ092), a pan-AKT inhibitor, in cancer (41). Low-dose miransertib reduces levels of phospho-AKT by about half in 83% of tissue samples from patients with Proteus syndrome (203) which is caused by a gain of function mutations in the AKT pathway (40). This inhibitor also demonstrates an anti-proliferative effect in fibroblasts isolated from PIK3CA-related overgrowth syndromes (204). Based on these findings, a phase I/II clinical trial is investigating the use of miransertib in PIK3CA-related overgrowth syndromes and other VAs.

There may be pitfalls in targeting specific mutations within the PI3K/AKT/mTOR or RAF/MEK/ERK pathways, as they interact via Ras (205) (Figure 2). *Kras* mutations which are common in non-small cell lung cancer (NSCLC), cause activation of the RAF/MEK/ERK pathway (206). This has led to the development of small molecule inhibitors that target MEK (207). However, these therapies are ineffective in NSCLC due to subsequent activation of the PI3K/AKT/mTOR pathway (208). Further, activating mutations in *PIK3CA* increase resistance to MEK inhibitors, and a mutation in *PTEN*, an inhibitor of PIK3CA, causes complete resistance (209). Therefore, a cautionary approach in targeting specific mutations in these pathways should be taken, as resistance may develop because alternative pathways are activated. It is important that research efforts continue to look broadly for an effective, safer and affordable treatment for VAs.

Even if existing targeted cancer therapies can be effectively repurposed for the treatment of VAs including overgrowth syndromes, the cost of such agents may be prohibitive. For example, the cost for a full-course of trastuzumab is USD50,000 (210). Another consideration is the potential long-term side effects of potentially life-long treatment for young patients with congenital anomalies. This underscores the need to better understand the etiology of this group of challenging conditions. The identification of the expression of the RAS by primitive populations in many types of VAs (Table 1) opens up an exciting prospect of novel therapeutic targeting of these primitive populations by manipulation of the RAS using low-cost, off-patent and commonly available oral medications.

## Conclusion

This review presents accumulating evidence demonstrating the presence of populations of cells that express stemness-associated markers in a growing number of vascular tumors and vascular malformations. As sirolimus targets cellular proliferation, survival and stemness via mTOR inhibition, its observed effect on some vascular tumors and vascular malformations, including overgrowth syndromes, may be attributed to its action on these primitive populations within these VAs. Gene mutations identified in VAs predominantly affect either the PI3K/AKT/mTOR or the Ras/RAF/MEK/ERK pathways. Existing cancer therapies that target these pathways open the possibility of repurposing these agents for the management of some of these challenging VAs. However, there are potential drawbacks, including the cost, potential long-term side effects in young patients, and the emergence of treatment resistance.

The discovery of cells that express stemness-associated markers in IH and the potential regulatory role of the RAS in its pathogenesis, underscore its spontaneous and accelerated involution induced by β-blockers and ACE inhibitors. Recent work demonstrating the effectiveness of targeting IH stem cells through inhibiting SOX18 using R-propranolol, may lead to more effective treatment of IH without the side effects of β-adrenergic blockade.

The observation of the expression of components of the RAS by populations of cells that also express stemness-associated markers in many types of VAs, opens up an exciting area of research. The stemness-associated markers expressed by cell populations and their cellular location within VAs are shown in Table 2. The expression of some or all transcription factors involved in generation of iPSCs by the cell populations in various types of VAs warrants further investigation including functional work to determine if they possess ESC properties. Studies are needed to determine whether mutations affecting the PI3K/AKT/mTOR or the Ras/RAF/MEK/ERK pathways, through their interaction with components of the RAS expressed on the primitive cells in VAs, could induce and/or maintain these cells in the primitive state. Further investigation into the precise interaction between the RAS and these pathways affected by the aforementioned mutations, and its effect on the stem cells may lead to improved understanding of the pathogenesis of these hitherto enigmatic conditions. This may provide immense opportunities for repurposing existing low-cost commonly available oral medications for the treatment of these challenging conditions.

**Table 2:** Expression and location of stemness-associated markers in vascular anomalies.

**Vascular anomaly**	**Stemness-associated markers**	**Location of stemness-associated markers**
Proliferating infantile hemangioma (50, 211)	OCT4, SSEA-4, pSTAT3, NANOG, SALL4, CD133	Endothelium of microvessels: all markers except NANOG. Cells within the interstitium: NANOG, SALL4, and CD133
Pyogenic granuloma (51)	OCT4, SOX2, pSTAT3, NANOG	Endothelium of microvessels: all markers. Cells within the interstitium: all markers except OCT4
Venous malformation (46)	NANOG, CD44, OCT4, SOX2, SALL4, CD44, pSTAT3	Endothelium of lesional vessels: all markers. Perivascular cells: all markers except OCT4 and SALL4
Verrucous venous malformation (47)	OCT4, brachyury	Endothelium of lesional vessels
Microcystic lymphatic malformation (45)	OCT4, NANOG, SOX2, KLF4, c-MYC	Endothelium of lesional vessels: all markers. Cells within the stroma: OCT4, c-MYC, and SOX2
Arterio-venous malformation (48)	OCT4, SOX2, KLF4, c-MYC	Endothelium and media of lesional vessels and cells within the stroma
Hypertrophic port-wine stain (49)	OCT4, SOX2, KLF4, c-MYC	Endothelium and media of lesional vessels and cells within the stroma

## Author Contributions

EK and LH drafted the manuscript. ST, PD, and SH critically revised the manuscript. All authors approved the manuscript.

## Conflict of Interest

The authors declare that the research was conducted in the absence of any commercial or financial relationships that could be construed as a potential conflict of interest.
